# Identification of rare genetic variation of NLRP1 gene in familial multiple sclerosis

**DOI:** 10.1038/s41598-017-03536-9

**Published:** 2017-06-16

**Authors:** Ales Maver, Polona Lavtar, Smiljana Ristić, Sanja Stopinšek, Saša Simčič, Keli Hočevar, Juraj Sepčić, Jelena Drulović, Tatjana Pekmezović, Ivana Novaković, Hodžić Alenka, Gorazd Rudolf, Saša Šega, Nada Starčević-Čizmarević, Anja Palandačić, Gordana Zamolo, Miljenko Kapović, Tina Likar, Borut Peterlin

**Affiliations:** 1 Clinical Institute of Medical Genetics, Slajmerjeva 3, University Medical Centre Ljubljana, Ljubljana, Slovenia; 2 Department of Biology and Medical Genetics, School of Medicine, University of Rijeka, Rijeka, Croatia; 3 Institute of Microbiology and Immunology, Faculty of Medicine, University of Ljubljana, Zaloška 4, 1000 Ljubljana, Slovenia; 4 Division of Neurology, University Medical Centre Ljubljana, Zaloška 2, 1000 Ljubljana, Slovenia; 5 Postgraduate Study, School of Medicine, University of Rijeka, Rijeka, Croatia; 6 Department of Pathology, School of medicine, University of Rijeka, Rijeka, Croatia; 7 Clinic of Neurology, CCS, Faculty of Medicine, University of Belgrade, Belgrade, Serbia; 8 Faculty of Medicine, University of Belgrade, Institute of Human Genetics, 26 Visegradska, Belgrade, Serbia; 9 Institute of Epidemiology, Faculty of Medicine, University of Belgrade, Belgrade, Serbia; 10 Natural History Museum Vienna, I. Zoological Department, Vienna, Austria

## Abstract

The genetic etiology and the contribution of rare genetic variation in multiple sclerosis (MS) has not yet been elucidated. Although familial forms of MS have been described, no convincing rare and penetrant variants have been reported to date. We aimed to characterize the contribution of rare genetic variation in familial and sporadic MS and have identified a family with two sibs affected by concomitant MS and malignant melanoma (MM). We performed whole exome sequencing in this primary family and 38 multiplex MS families and 44 sporadic MS cases and performed transcriptional and immunologic assessment of the identified variants. We identified a potentially causative homozygous missense variant in NLRP1 gene (Gly587Ser) in the primary family. Further possibly pathogenic NLRP1 variants were identified in the expanded cohort of patients. Stimulation of peripheral blood mononuclear cells from MS patients with putatively pathogenic NLRP1 variants showed an increase in IL-1B gene expression and active cytokine IL-1β production, as well as global activation of NLRP1-driven immunologic pathways. We report a novel familial association of MS and MM, and propose a possible underlying genetic basis in NLRP1 gene. Furthermore, we provide initial evidence of the broader implications of NLRP1-related pathway dysfunction in MS.

## Introduction

Multiple sclerosis (MS) is a chronic inflammatory disease of the central nervous system, which leads to widespread focal lesions of primary demyelination with variable axonal, neuronal and astroglia injury. Despite extensive research, key aspects of multiple sclerosis etiology and pathogenesis still remain unresolved. It is considered that multiple and complex interactions between environment and genetic background lead to the manifestation of the disease. Nevertheless, neither environmental triggers nor highly penetrant genetic predisposition genes have yet been discovered. Familial contribution to MS etiology is well recognized and many cases of families with apparent monogenic inheritance have been reported However, in contrast to several other complex neurological disorders no highly penetrant genetic variants have been described in MS yet. Exome sequencing of several MS families with multiple affected members has identified genetic variants in two genes, CYP27B1 and TYK2 with the modest effect on MS risk^1, 2^. In a recently published study, a pathogenic variant in NR1H3 gene (p.Arg415Gln), has been reported in two independent families with an autosomal dominant pattern of inheritance^3^. In the reported two NR1H3 families, segregation with the disease was not complete and the same variant was also present among presumably healthy controls of the general population, making this association uncertain, based on the available evidence.

In our study, we used whole-exome sequencing to identify a putative genetic cause in the nuclear family, where MS cosegregated with malignant melanoma (MM). In the nuclear family, we used exome sequencing in combination with homozygosity mapping to uncover a possibe causative homozygous NLRP1 gene variant in affected cases. To date, the association with MS and MM has only been reported in epidemiological surveys as comorbidities^4^, but evidence of familial co-occurrence and shared etiological origin of these two diseases has not been reported. Interestingly, studies have reported genetic association of variation in NLRP1 gene and susceptibility to MM, and there are recent reports supporting the role of NLRP1 in driving tumorigenic processes in malignant melanoma^5^.

We followed the initial finding by exploring the association of NLRP1 and MS in a wider set of patients with familial and sporadic MS. Based on these findings, we provide initial evidence of the broader implications of NLRP1-related pathway dysfunction in familial and sporadic MS.

## Results

### Identification of the primary family with concurrent multiple sclerosis and malignant melanoma

**Figure 1:**
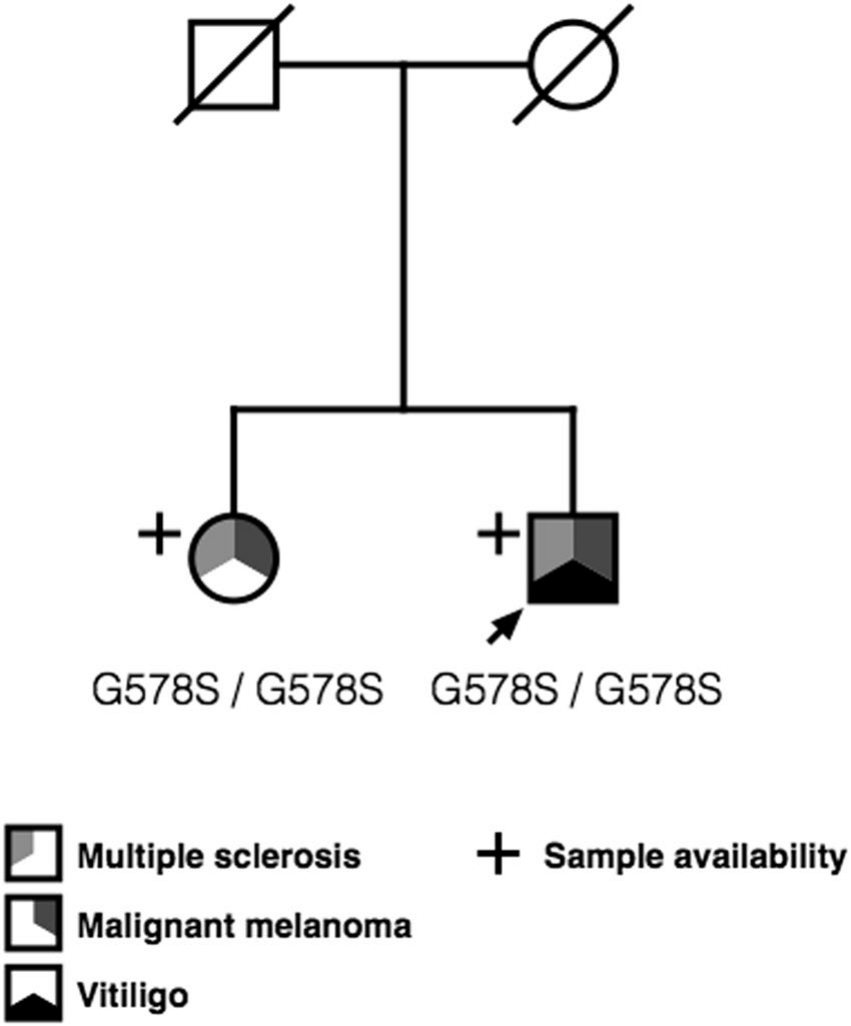
Family segregation patterns and mutations detected in the MSMM Family. The family includes two probands (male MSMM001 and female MSMM002), concurrently affected by multiple sclerosis and malignant melanoma. Both probands were homozygous for a rare Gly578Ser predicted pathogenic variant in the NLRP1 gene.

We identified a family with two sibs concurrently affected by multiple sclerosis and malignant melanoma (MSMM, Fig. 1).

In the male proband (MSMM001) fatigue and weakness of the lower extremities started at age 37. However, multiple sclerosis was first suspected at the age of 41, after an MRI scan was performed in diagnostic evaluation of a traumatic vertebral fracture. The scan has shown signs of bilateral demyelinating lesions in periventricular white matter. At the age of 43, the patient was hospitalized because of diplopia and blurred vision dominating in the right eye. Mild ataxia and spastic paraparesis were also noted. Cerebrospinal fluid analysis showed intrathecal IgG synthesis and presence of oligoclonal bands. At the age of 50, the patient presented with left axilla lymphadenopathy, which was treated surgically. Perioperative biopsy showed the presence of S-100 positive atypical melanocytes, suggestive of malignant melanoma, and accordingly oncologic treatment was initiated. At the age of 57, the patient developed symptoms of vitiligo progressing for 5 months. This was concurrent with development of massiveright axilla lymphadenopathy. The histologic evaluation confirmed presence of malignant melanoma cells and oncologic treatment was repeated. The most recent neurological evaluation was performed at the age of 62 demonstrating the progression of MS (EDSS 4.5).

Proband’s sister (MSMM002) presented at the age of 40 with initial symptoms of MS, which included vertigo and right-sided hand and leg weakness. After quick remission, symptoms of blurred vision in the left eye and hand paresthesia appeared at the age of 42. MRI scan showed an hyper-intense lesion of the pons and multiple periventricular white matter lesions. Cerebrospinal fluid analysis showed intrathecal IgG synthesis and the presence of oligoclonal bands. At the age of 55 she was examined for suspicious nevus of the skin in the left hypochondriac region of abdomen. Biopsy of the nevus confirmed the presence of malignant melanoma. The patient deteriorated severely to the age of 59 and demonstrated severe spastic paraparesis, mixed ataxia, urinary incontinence and cognitive impairment (EDSS 9.0). She died at the age of 63 due to metastatic malignant melanoma.

In both reported cases, diagnosis of MS was established in accordance with McDonald criteria^6^. Both patients were treated with pulses of high-dose methylprednisolone at the time and both responded favorably to this treatment. No immunomodulating or other specific treatment was prescribed apart from symptomatic treatment of disease manifestations.

The striking co-occurrence of the two presenting diseases directed us towards a search for potential rare genetic variants related to the observed phenotype in the presenting family. The parents of the two patients were not diagnosed with either multiple sclerosis or malignant melanoma. The inheritance pattern observed in the family suggested an autosomal recessive mode of inheritance.

### Exome sequencing in the MSMM family

We performed whole exome sequencing in the two sibs concurrently affected by MS and MM. We initially focused the analysis to on variants in genes known to cause familial malignant melanoma, but we did not identify any clearly causative variation in these genes (BAP1, CDK4, CDKN2A, MC1R, MITF and PTEN). Afterwards we performed an analysis of all identified variants detected by exome sequencing, followed by selection of variants with predicted functional impact, low population frequency and selection of variants adhering to the segregation pattern in the primary family. This strategy has singled out a rare homozygous variant in NLRP1 gene, introducing an amino-acid change of glycine at protein position 578 to serine. The variant was present in the homozygous state in both patients with concurrent MS and MM phenotype. Furthermore, homozygosity mapping in the two probands revealed the presence of six contiguous blocks of homozygous SNPs, shared by both affected individuals (Supplementary Figure 1), which narrowed the initial search to variants within these regions. Analysis and filtering for rare sequence variants with predicted functional effect in the identified blocks of homozygosity has singled out a homozygous sequence variant in the NLRP1 gene within chromosome 17 block of homozygosity. Analysis of coverage depth in the region of the identified variant did not reveal presence of gene deletions that would mimic the homozygous status of the identified regions.

NLRP1 gene codes for the key protein component of the inflammasome complex, associated to vitiligo-associated multiple autoimmune disease (OMIM:193200), and other predominantly immune andmalignant disorders^7, 8^.

**Figure 2:**
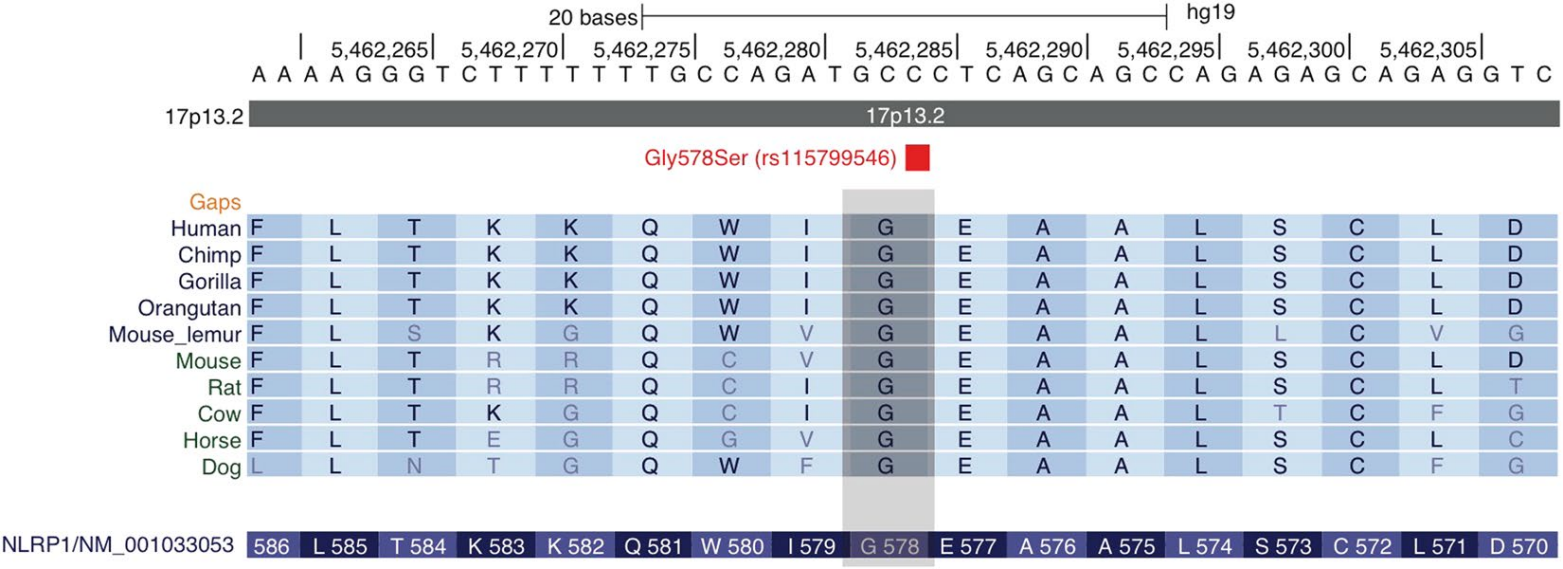
Genomic context and evolutionary conservation of NLRP1 homozygous variant identified in primary NLRP1 family 1 (Fig. 2). Variant affects amino acid position 578 in NLRP1 gene evolutionarily invariant in all mammal datasets in the UCSC Genome Browser conservation chain.

Further assessment of the identified variant has shown that it is located in the key functional effector domain of the NLRP1-encoded protein – NACHT triphosphatase domain, which mediates downstream caspase activation. Considerable evolutionary conservation of the affected amino acid was also observed (Fig. 2) and functional prediction algorithms (SIFT, Polyphen-2) classified the variant as pathogenic.

Genotyping of the identified variant in a 960 population control samples showed absence of the variant in a background population. The variant was also absent in UK10K and GoNL control populations. Carrier frequency in 60,657 individuals from heterogeneous populations included in the ExAC project has shown that the frequency of the variant in populations of European descent was exceedingly low (1.5∙10^−5^) and the variant was absent in European Finnish, East Asian and Latino populations. The variant was also observed in African populations at low frequency of 4.0∙10^−3^. No homozygous individuals have been reported in any of the population screening projects surveyed.

### Sequencing of NLRP1 gene in an expanded cohort of familial and sporadic MS cases

We expanded the search for further NLRP1 associated examples of MS presenting as either familial or sporadic cases by exome sequencing and targeted analysis of NLRP1 in the sequencing data. We performed these analyses in whole exome sequencing data from probands of 38 multiplex MS families, in 44 sporadic MS cases and 92 population-matched controls.

In familial cases of MS we identified five potentially damaging variants that were absent in the set of control individuals from the background population. One notable case was a frameshift Asn864ThrfsX4 variant identified in a familial MS case and absent from all control populations investigated (Table 1). The variant is predicted to result in the introduction of premature stop codon 4 aminoacid positions downstream of the variant site, likely representing a loss of function variation. The course of MS in five patients carrying these rare NLRP1 variants was variable and included relapsing-remitting, primary progressive and benign courses. Interestingly, the case with the frameshift NLRP1 variant had an early reported onset of the disease with symptoms presenting at 21 years of age with the disease following a relapsing-remitting pattern.

**Table 1:** Rare, possibly pathogenic variants in the NLRP1 gene are presented, along with their functional impact, frequency in ExAC populations of European descent and theoretical predictions of pathogenicity.

Chr	Position	Ref	Alt	Variant class	Transcript change (NM_033004.3)	Protein change	ExAC MAF	Background population MAF*	Polyphen2	SIFT
17	5445285	T	—	Frameshift	c.2591delA	Asn864fsX4	0,000%	0,000%	/	/
17	5424908	C	T	Missense	c.3719G > A	Arg1240His	0,003%	0,000%	B	T
17	5485999	G	A	Missense	c.439C > T	Arg147Cys	0,010%	0.004%	B	T
17	5461960	T	C	Missense	c.2056A > G	Met686Val	0,039%	0,000%	P	D
17	5463093	C	T	Missense	c.923G > A	Arg308Gln	0,110%	0,009%	B	T
17	5445243	G	A	Missense	c.2633C > T	Thr878Met	4,429%	7,500%	B	T
17	5463279	G	C	Missense	c.737C > G	Thr246Ser	4,409%	7,200%	B	T
17	5437285	G	A	Missense	c.2984C > T	Thr995Ile	4,772%	7,200%	P	T
17	5433966	T	C	Missense	c.3355A > G	Met1119Val	4,808%	7,600%	B	T
17	5424906	C	G	Missense	c.3721G > C	Val1241Leu	4,875%	7,500%	B	T

All the listed variants were identified in cases with familial MS, with the frequency in control populations below 5%. *Minor allele frequency (MAF) in the population-matched background population of 1000 population matched exomes.

In 3 individuals with familial MS (7.9%), 7 individuals with sporadic MS (15.9%) and 9 controls (9.8%), we also identified a set of co-occurring variants: Thr246Ser, Thr878Met, Thr995Ile, Met1119Val in Val1241Leu which corresponded to the autoimmunity-associated haplotype (H3) containing a set of rare missense variants in NLRP1, previously identified and associated with autoimmune diseases (Table 1 and Supplementary Figure 2)^8^. Although the frequency in the sporadic MS population was increased in comparison to the control population this difference failed to reach statistical significance (Chi-square test P = 0.21).

We also performed a statistical test of mutational burden in NLRP1 gene, including variants with moderate or high functional impact and below 5% in ExAC project in the analysis. We could not demonstrate an overall excess of potentially altering variants in the NLRP1 gene in familial MS cases (P = 0.92) when comparing them to control subjects. There was an excess of missense variants in the sporadic MS groups in comparison to control cases (P < 0.0001), likely due to a high overall number of missense variants belonging to the H3 haplotype.

### Expression profiling of stimulated PBMCs

To characterize the functional impact of the variants in NLRP1 gene and their possible effect on the immune response in patients with MS we performed transcriptional and immunologic analyses. We included the patient from the primary family concomittantly affected with MS and MM, and also two patients from a family carrying the frameshift NLRP1 variant and in two cases from a family with Arg147Cys variant.

**Figure 3:**
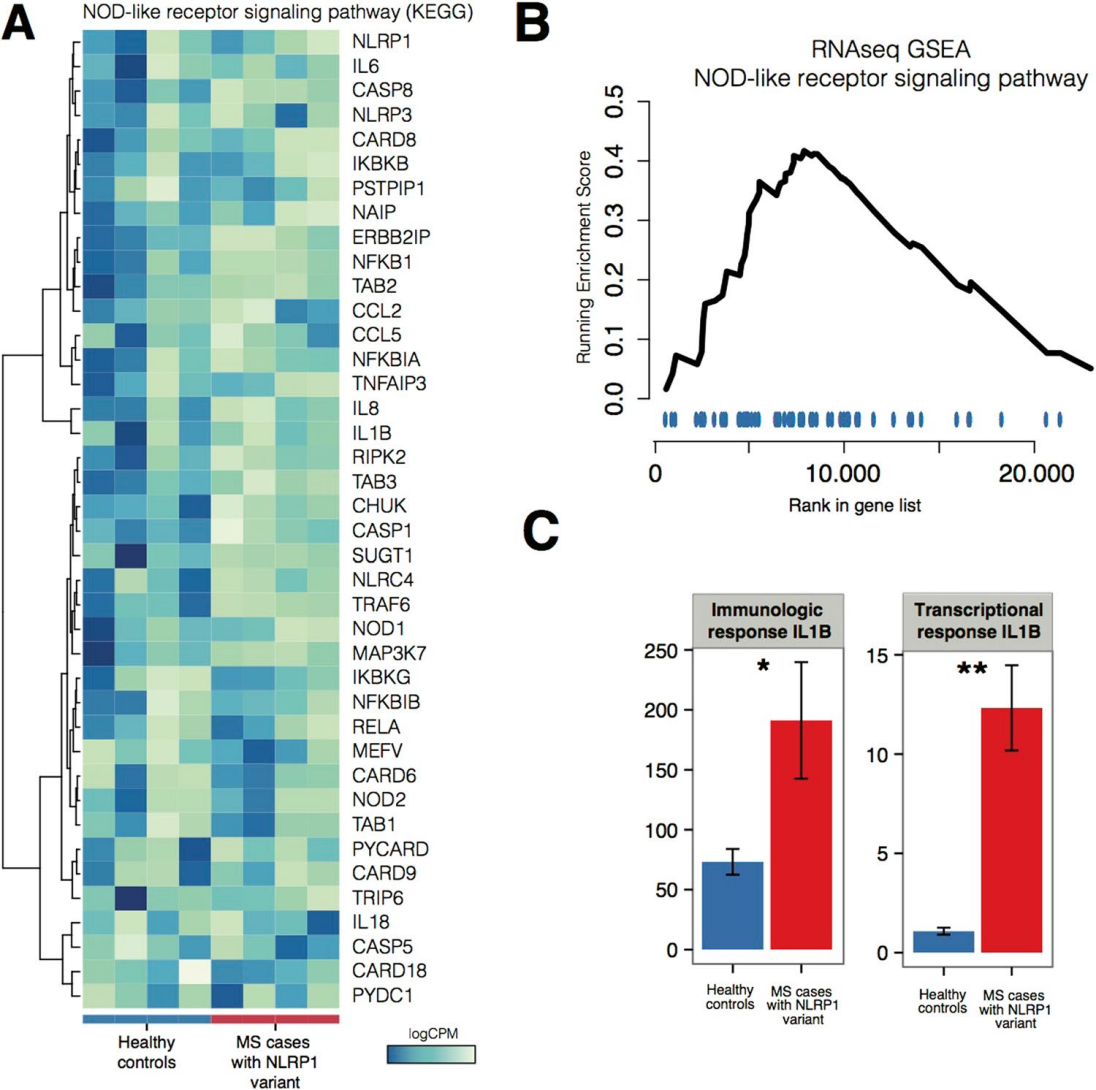
Immunological and transcriptional characterization of PBMCs response to NLRP1 pathway stimulation in MS patients carrying putatively pathogenic variants in NLRP1 gene in comparison to healthy controls. (**A**) depicts results of RNAseq transcriptional profiling for a selection of genes belonging to broad inflammasome pathway as defined by KEGG NOD-like receptor pathway. A tendency towards upregulation of genes in the pathway is present and the recapitulated with GSEA pathway analysis, presented in (**B**) showing a tendency of the pathway towards upregulation. (**C**) depicts characterization of IL-1β as the main effector of NLRP1 pathway activation. We were able to both validate the increased expression of IL1B detected in RNAseq and to demonstrate increased production of IL-1β. The specificity of findings for IL-1β was demonstrated by measuring TNF-α production in the same cell culture supernatants (data not shown)^8^. There was no significant difference in the TNF-α secretion between PBMCs of MS patients with mutations in NLRP1 gene and healthy subjects. The * and ** annotations in (**C**) denote significance attained at threshold P = 0.05 and P = 0.01, respectively. The intervals correspond to standard error measure (SEM) of the measurements.

Using transcriptome sequencing of stimulated PMBCs we were able to demonstrate an overall upregulation of inflammasome related genes in the NOD-like receptor KEGG pathway in patients carrying putatively causative variants in NLRP1 (with q-value of 0.0030, Fig. 3A,B).

Expression profiling of genes after stimulation of NLRP1 pathway has shown significant differences in expression of downstream effector genes. Specifically, we detected an increased response of IL1B gene expression (p = 0.00046, Fig. 3C) when comparing MS mutation carriers to healthy controls.

We also surveyed the expression of effector pathways downstream of NLRP1 stimulation and detected an increased expression of genes belonging to several effector pathways including NFKB1, JUN AND p38 pathway (Supplementary Figure 3).

## Discussion

In the present study we report a novel familial association of MS and MM, and propose a possible underlying genetic basis in NLRP1 gene. Furthermore, we provide initial evidence of the broader implications of NLRP1-related pathway dysfunction in familial and sporadic MS.

In the family with familial association of MS and MM, exome sequencing in combination with homozygosity mapping led us to identification of a rare homozygous sequence variant (Gly587Ser) in the NLRP1 gene predicted to affect function, suggesting an autosomal recessive genetic predisposition. The variant encompasses a change of a highly evolutionary conserved amino acid residue of the NACHT domain which is required for proper NLRP1 inflammasome oligomerization and assembly^9^. Notably, pathologic variants in NACHT domain of NLRP3 inflammasome counterpart have already been shown to cause inherited NLRP3-related autoinflammatory syndromes^10, 11^. The role of NLRP1 in the pathogenesis of MM is supported by reported genetic association^7^ and constitutive activation of NLRP1 inflammasome leading to persistently increased production of downstream mediators (Caspase-1 and IL-1β) in malignant melanoma tissue^12^. Multiple sclerosis and malignant melanoma have previously only been associated in epidemiological studies as comorbid conditions^4^, yet evidence of familial co-ocurrence and shared etiological origin of these two diseases has not been reported to date. Whilst there have been reports linking specific immunomodulating drugs currently used in treatment of MS and increased susceptibility to MS, both affected sibs of the primary family affected by MS and MM were not treated with these or similar therapies.

Furthermore, we found several additional possibly associated sequence variants in the NLRP1 gene, one novel frameshift variant (Asn864ThrfsTer4) and 4 rare missense NLRP1 variants in distinct MS families. We also identified the presence of H3 haplotype associated with immune disease susceptibility in MS patients and controls, but statistical comparisons failed to reveal difference in frequency between the groups of familial MS, sporadic MS and control subjects. Interestingly, when the sum of all moderate to high impact variants with frequency below 5% was taken into consideration with burden analysis we could observe a significant excess of moderate to high impact variants in patients with sporadic MS.

Functional immunologic studies in selected patients with putatively pathogenic NLRP1 variants have shown that stimulation of PBMCs in variant carriers resulted in overexpression of downstream effector IL-1β in patients with NLRP1. Whole transcriptome profiling of PBMCs further showed overexpression of complete NOD-like receptor pathway and components of downstream NFKB, JNK and p38 pathways which were previously implicated in the MS pathology^13–15^.

The role of the IL-1β has been implicated in a variety of inflammatory and neurodegenerative processes occurring in multiple sclerosis. There is evidence that IL-1β induces trans-endothelial migration of activated leukocytes across the blood-brain barrier of the central nervous system^16^, exacerbates central neuroinflammation independently of the blood-brain barrier integrity^15^ and facilitates T helper 17 cell induction^17^. IL-1β is present in the CNS lesions in MS^18^ and its concentration is increased in CSF fluid of patients with MS^19^ and correlates with cortical pathology load^20^.

Animal studies lend further support to the role of NLRP1 in susceptibility to multiple sclerosis. In a recent study by Masters *et al*., mice carrying a semi-dominant pathogenic variant (Q593P) in Nlrp1a ortholog of NLRP1 were shown to display a distinct phenotype of multisystemic inflammatory disease that also prominently affected the central nervous system^21^. While the mouse models with Nlrp1a functional variants usually presented with notably altered immunological profiles, our patients’ immunological profiles were within physiological range in the resting state. It is thus possible that the identified variants exert abnormal stimuli upon external provocation by either infectious or other environmental provocations.

Inflammasomes constitute molecular sensor systems activated by environmental stimuli such as cellular infection (viral, bacterial and prokaryotic) or other stressors, and as such present an interface to a variety of exposures triggering the cellular inflammatory response^22^. There is an increasing body of evidence corroborating the role of viral infection, UV light exposure, smoking and other stimuli, known to increase the susceptibility to MS^23, 24^.

Based on our results we hypothesize that the combination of genetic predisposition for increased stimulation of NLRP1 pathway to various environmental exposures may contribute to compromised blood-brain barrier integrity, recurrent active demyelination and neurodegeneration observed in MS.

Evidence for the role of inflammasome in the pathogenesis expands the field of potential therapeutic intervention in MS. Biological treatments targeting IL-1 signaling have been approved for treatment of various auto-inflammatory and autoimmune disorders^25^. Specifically, human monoclonal antibodies against IL-1β (canakinumab) have shown clinical benefit in patients with NLRP3-related disorders, while clinical trials for use in autoimmune disorders are already underway^25^.

In conclusion, we present a novel familial association of MS and MM and identify a possible genetic basis for this association in the NLRP1 gene. We identified additional candidate variants in NLRP1 gene in an expanded cohort of patients with familial MS. The role of human inflammasome at the interface between genetic predisposition and environment may offer further insight in the pathogenesis, prevention and treatment of multiple sclerosis.

## Methods

### Ethical statement

The study was performed in accordance with the principles stated in the Declaration of Helsinki. The study on hereditary risk factors in multiple sclerosis was approved by the National Medical Ethics Committee (90/08/12). All participants gave informed written consent to participate in the study.

### Genetic and functional analyses

We performed whole exome sequencing in affected probands of the primary family (Family 1, with concurrent multiple sclerosis and malignant melanoma), followed by homozygosity mapping and characterization of discovered candidate variants, which singled out putatively pathogenic variation in NLRP1 gene. We further expanded the search for pathogenic NLRP1 variants in an expanded set of 38 patients with familial MS and 58 patients with sporadic MS and identified two further MS families carrying potentially pathologic NLRP1 variants. To functionally characterize NLRP1 genetic variants we performed targeted immunologic stimulation of NLRP1 pathway in patients with putatively pathogenic NLRP1 variants and healthy controls. In stimulated PBMCs we measured the activity of downstream NLRP1 effectors and performed whole transcriptome profiling using RNAseq followed by targeted quantitative PCR validation of identified gene expression alterations.

### Exome sequencing

Aiming to survey the presence of potential causative genetic variation in the MSMM family we performed exome sequencing of both affected probands in the primary family (Fig. 1). The sequencing was performed after whole human exome target capture with Agilent SureSelect v4 capture kit. Sequencing of enriched shotgun library was performed on HiSeq 2000 platform in the paired-end mode. We reached median on target coverage exceeding 50x in both affected sibs (denoted MSMM001 and MSMM002), assuring sufficient variant detection rate in both cases. The detailed variant filtration and exome data analysis strategies are presented in Supplementary Methods.

We also performed homozygosity mapping in exome data, utilizing the HomozygosityMapper tool (available at www.homozygositymapper.org ^26^). Only the variants in regions covered at 50x depth or more were included in this analysis and minimal block sizes was set at 20 variants, while leaving other parameters in their default state.

### Population screening of identified variants in general population

Screening for sequence variants identified in MS patients was performed in 960 population control samples using custom designed KASPAR genotyping assays (LGC) using ABI 3000 Sequence Detection System (Applied Biosystems) as previously described and in accordance with manufacturers recommendations^27^.

### Exome sequencing in an expanded group of MS patients

We have selected further 96 samples with MS for targeted interrogation of coding sequence variants in NLRP1 genes with Sanger sequencing. Of these, 38 samples originated from index cases in multiplex families with at least two affected members while the remaining 58 cases were sporadic. As with sequencing in the primary MSMM family, exome sequencing was performed using Illumina Nextera Coding Exome pull-out protocol followed by sequencing on Illumina HiSeq 2500 and bioinformatics analysis using in-house pipelines, as described above.

### Isolation and stimulation of PBMCs

Detailed methods for isolation and stimulation of PBMCs are presented in the Supplementary Materials.

### Immune status of subjects

Detailed methods for determination of immune status of the patients are presented in the Supplementary Materials.

### RNAseq and quantitative PCR experiments

Detailed methods for RNAseq and qPCR experiments are presented in the Supplementary Materials.

## Electronic supplementary material

Supplementary Methods
