# Supplementary material for: Identification of rare genetic variation of NLRP1 gene in familial multiple sclerosis
Source: Sci Rep. 2017 Jun 16;7:3715. doi: 10.1038/s41598-017-03536-9 (PMC5473861; doi:10.1038/s41598-017-03536-9)
Supplement: Supplementary file 1 — Supplementary Methods [file 41598_2017_3536_MOESM1_ESM.doc]

**Supplementary information**

**Identification of rare genetic variation of NLRP1 gene in familial multiple sclerosis**

Maver Ales^1^, Lavtar Polona^1^, Ristič Smiljana^2^, Stopinjšek Sanja^3^, Simčič Saša^3^, Hočevar Keli^1^, Sepčić Juraj^5^, Drulović Jelena^7^, Pekmezović Tatjana^9^, Novaković Ivana^8^, Hodžić Alenka^1^, Rudolf Gorazd^1^, Šega Saša^4^, Starčević-ČizmarevićNada^2^, Palandačić Anja^1^, Zamolo Gordana^6^, Kapović Miljenko^2^, Likar Tina^1^, Peterlin Borut^1§^

**Supplementary Methods**

**Exome sequencing data analysis**

Results generated were analysed using the custom pipeline established at the Centre for Mendelian Genomics, Ljubljana, Slovenia. After demultiplexing and trimming of adaptor sequences, reads were assessed in terms of sequencing quality and those falling below the required quality threshold were excluded from subsequent analyses. Reads were mapped to UCSC hg19 reference genome assembly using bwa short-read aligner. Realignment around indels, base quality score recalibration and variant quality score recalibration were performed in accordance with GATK best practice guidelines. Only variants passing the GATK-assigned quality criteria above 50.0 were regarded for down-stream analyses.

Variant effect prediction was done using ANNOVAR and snpEff toolsets and annotated using population based annotations (1000genomes, ESP6500, UK10K, cg69 and our in-house variant frequency database), pathogenicity prediction utilities (SIFT, PolyPhen2 and MutationAssessor) as well as evolutionary conservation measures (GERP).

**Immune status of the patients**

The immune status of patients with MS and healthy subjects was evaluated by the BD Multitest 6-color TBNK Reagent with BD Trucount tubes, to determine the percentage and absolute counts of T, B, and natural killer (NK) cells as well as the CD4^+^ and CD8^+^ subpopulations of T cells in peripheral blood, and by the BD Simultest CD3/Anti-HLA-DR for enumerating percentages of mature human activated T cells in erythrocyte-lysed whole blood (BD Biosciences, USA). Samples were processed by FACSCanto II flow cytometer (BD Biosciences, USA) and data analysed by FACSCanto clinical software. The immune status of all subjects included in the study was normal (data not shown).

**RNAsequencing experiments**

Total RNA sequencing in stimulated PBMCs from 4 MS patients with putatively pathogenic NLRP1 variants and 4 healthy controls was performed on RNA samples, isolated from estimated 1 million cells using Qiagen RNeasy Mini isolation kit, according to manufacturers protocol. Libraries for RNA sequencing were prepared using TruSeq RNA stranded library preparation kit (Illumina), including RiboZero bead-based rRNA depletion protocol. Sequencing of 8 libraries pooled in equimolar concentrations was performed on Illumina MiSeq sequencing system in 2x150 sequencing cycles using paired-end sequencing mode. After initial read quality filtering and demultiplexing using MiSeq Real Time Analysis (RTA) software, alignments to human reference genome (hg19) were performed bowtie and tophat alignment softwares. Transcript abundances were then estimated using read counts function in Subread package for R [^1^](#_ENREF_1), followed by intersample normalization by variance modeling at the observational level (voom) approach implemented in limma Bioconductor package [^2^](#_ENREF_2). Differences in gene expression were estimated using linear modeling and significance estimation procedures in limma.

**Quantitative real-time polymerase chain reaction**

cDNA was prepared from 300 ng RNA using the SuperScript Vilo reverse transcriptase (Invitrogen, Carlsbad, USA) according to manufacturer’s instructions. qPCR was performed using TaqMan® SYBRgreen chemistry (Applied Biosystems, Foster City, USA), using pre-tested PrimerBank primer sequences. The list of primers used in qPCR experiment is provided in Supplementary Table 1. All qPCR reactions were performed in triplicates in a 11.1 μl final volume reactions, consisting of 1.0 μl of diluted cDNA sample, 5.5 µL of SYBR® Green, 4.4 µL of nuclease free water and 0.1 µL of each primer. The measurements were performed using ABI Prism 7000 Sequence detection system (Applied Biosystems). Thermal cycling conditions were as follows: an initial step at 95°C for 10 min and 30 cycles of denaturation at 95°C for 15s, annealing at 57°C for 30s and polymerization at 72°C for 30s. The threshold cycle (Ct) values were then determined for each assay and data were normalized to internal control beta-actin (ACTB gene) that was co-ran with each sample.

**Isolation and stimulation of PBMCs**

Peripheral blood mononuclear cells (PBMCs) from MS patients with mutations in NLRP1 gene (n = 5) and healthy subjects (n = 6) were isolated from freshly drawn venous blood with EDTA by density gradient centrifugation with Ficoll-Paque™ (GE Healthcare, UK). All reagents were commercially obtained from Sigma-Aldrich Corp. (USA), unless otherwise stated. For the *in vitro* stimulation of PBMCs we have modified a model which has been described previously[^3^](#_ENREF_3). The cells were cultured in the RPMI-1640 medium with 25 mM HEPES, supplemented with 100 U/ml penicillin, 100 μg/ml streptomycin, 2 mM L-glutamine and 10% heat-inactivated human serum. The 5×10^5^ cells were seeded in 24-well culture plates (Corning Costar, USA) with medium alone or stimulated with MDP (10 µg/ml) at 37 °C in a humidified atmosphere of 5% CO_2_ in air. After 18 hours of incubation, ATP (1 mM) or medium were added for an additional 1 hour, since oligomerisation of NLRP1 and activation of caspase-1 in the NLRP1 inflammasome is a two-step mechanism[^4^](#_ENREF_4). The cell culture supernatants were then collected and stored at −80 °C before further analysis. The concentrations of cytokines in cell culture supernatants were measured in pg/ml using commercially available ELISA kits. A mature form of IL-1β was measured by the Human IL-1β ELISA kit and TNF-α by the Human TNF-α ELISA kit (Thermo Scientific Pierce, USA). To determine statistically significant differences in cytokine production between the two groups of subjects, we used the nonparametric Mann-Whitney test. P values less than 0.05 were considered statistically significant.

**Supplementary Figures**

**Supplementary Figure 1**

Homozygosity mapping showed 6 shared regions of homozygosity in both affected probands, including the region on chromosome 17, containing homozygous variant in the NLRP1 gene.

**
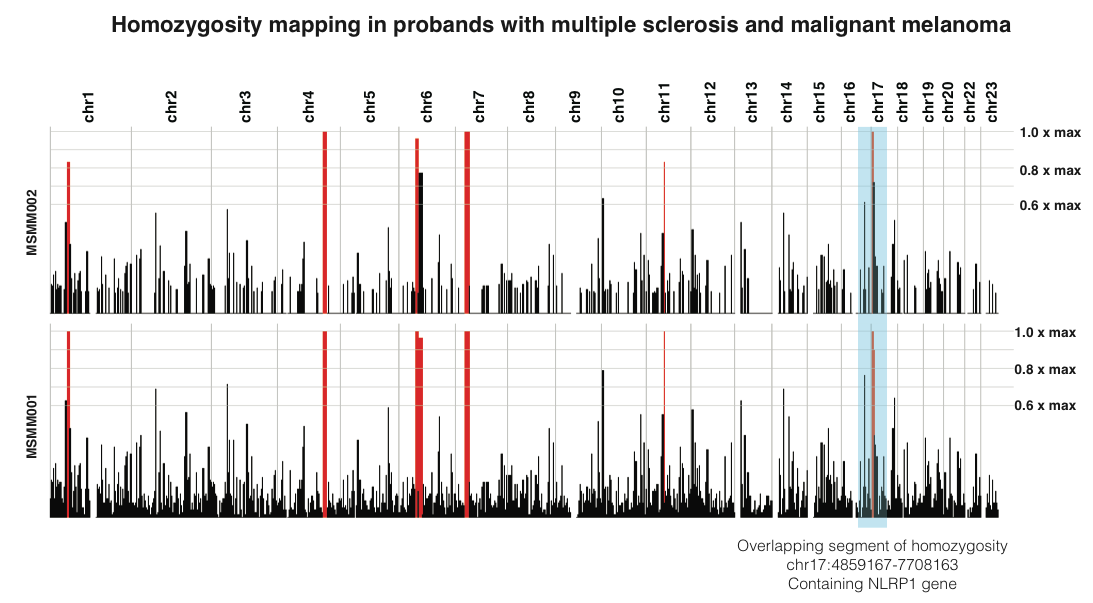
**

**Supplementary Figure 2**

Haplotype structure determined in 396 Slovenian controls. The largest block of variants defines 5 unique haplotype, in accordance with Levanodowski et al. 2013.


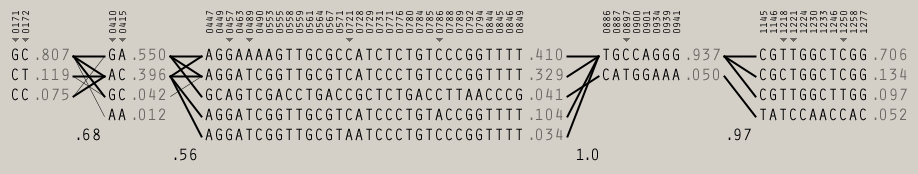
**
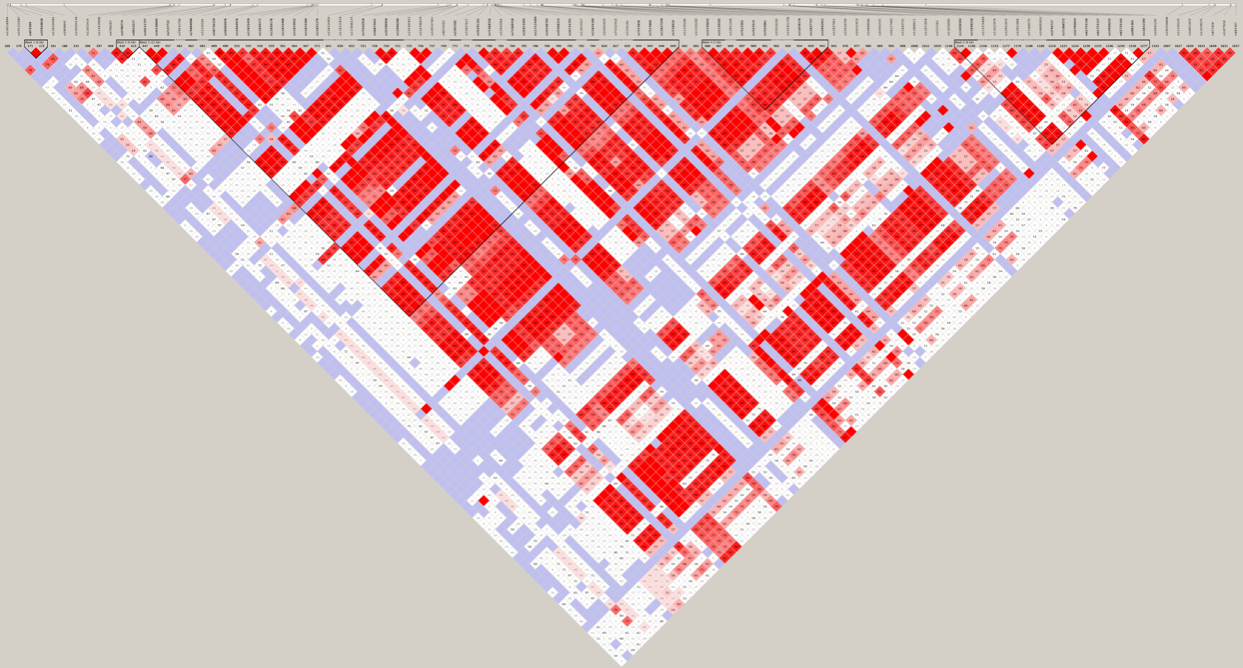
**

**Supplementary Table 1**

Primer sequences utilized for quantitative PCR profiling of selected transcripts.

| **Primer name** | **Primer sequence** |
| --- | --- |
| IL18_F | TCTTCATTGACCAAGGAAATCGG |
| IL18_R | TCCGGGGTGCATTATCTCTAC |
| CASP1_F | TTTCCGCAAGGTTCGATTTTCA |
| CASP1_R | GGCATCTGCGCTCTACCATC |
| CASP5_F | TCACCTGCCTGCAAGGAATG |
| CASP5_R | TCTTTTCGTCAACCACAGTGTAG |
| NLRP1_F | GCAGTGCTAATGCCCTGGAT |
| NLRP1_R | GAGCTTGGTAGAGGAGTGAGG |
| IL1B_F | ATGATGGCTTATTACAGTGGCAA |
| IL1B_R | GTCGGAGATTCGTAGCTGGA |

**Supplementary references**

1. Liao Y, Smyth GK and Shi W. The Subread aligner: fast, accurate and scalable read mapping by seed-and-vote. *Nucleic acids research*. 2013; 41: e108.

2. Law CW, Chen Y, Shi W and Smyth GK. voom: Precision weights unlock linear model analysis tools for RNA-seq read counts. *Genome biology*. 2014; 15: R29.

3. Simcic S, Wraber B, Sollner M, Urleb U and Gobec S. Modulation of tumour necrosis factor production with desmuramyldipeptide analogues. *Pflugers Archiv : European journal of physiology*. 2000; 440: R64-6.

4. Faustin B, Chen Y, Zhai D, et al. Mechanism of Bcl-2 and Bcl-X(L) inhibition of NLRP1 inflammasome: loop domain-dependent suppression of ATP binding and oligomerization. *Proceedings of the National Academy of Sciences of the United States of America*. 2009; 106: 3935-40.
